# Prevalence of Ankyloglossia in Portuguese Newborns and Its Effect on Exclusive Breastfeeding and Maternal Nipple Pain During Postpartum Hospitalization

**DOI:** 10.1016/j.jpedcp.2025.200195

**Published:** 2025-11-13

**Authors:** J. Morgado Dias, J. Abanto, J. Correia Pinto, C. Areias, H. Soares

**Affiliations:** 1 Faculty of Dental Medicine, Pediatric Dentistry Department, Oporto University, Porto, Portugal; 2 Paulista Association of Dental Surgeons, Pediatric Dentistry Department, School of Dentistry, São Paulo, Brazil; 3 Faculty of Dentistry, Pediatric Dentistry Department, Catalonia International University, Barcelona, Spain; 4 Department of Pediatric Surgery, Braga Hospital, Braga, Portugal; 5 School of Medicine, University of Minho, Minho, Portugal; 6 RISE: Health, I&D Unit, Faculty of Dental Medicine, University of Porto, Porto, Portugal; 7 Department of Neonatology, São João Hospital, Porto, Portugal; 8 CINTESIS@RISE, Faculty of Medicine, University of Porto, Porto, Portugal

**Keywords:** Ankyloglossia, breastfeeding, lingual frenulum, tongue-tie

## Abstract

**Objectives:**

To evaluate the association between ankyloglossia and breastfeeding outcomes, particularly exclusive breastfeeding (EBF) and maternal nipple pain during the immediate postpartum period and to estimate the prevalence of ankyloglossia in a population of Portuguese newborns.

**Study design:**

This cross-sectional study assessed 501 newborns within the first 5 days of life at Centro Hospitalar Universitário de São João, Oporto, Portugal, before hospital discharge. Ankyloglossia was diagnosed by a single examiner using the Assessment Tool for Lingual Frenulum Function protocol. Data on breastfeeding practices and maternal nipple pain were collected. Poisson regression analysis was used to evaluate associations.

**Results:**

The prevalence of ankyloglossia among newborns was 13.8%. During hospitalization, 71.0% of newborns were exclusively breastfed, and 28.4% of mothers reported nipple pain during breastfeeding. There was no statistically significant association between ankyloglossia and EBF rates during hospitalization. However, mothers of newborns diagnosed with ankyloglossia had a significantly higher probability of experiencing nipple pain during breastfeeding (prevalence ratio, 1.51; 95% CI, 1.11-2.06).

**Conclusions:**

Although ankyloglossia was not associated with lower EBF rates during the hospital stay, it was significantly linked with increased maternal nipple pain during breastfeeding. These findings suggest that, although ankyloglossia may not hinder EBF in the immediate postpartum period, it can contribute to maternal discomfort and potentially adversely affect the breastfeeding experience.

Breastfeeding and breast milk continue to be the first choice for newborns and infants.^1^, ^2^, ^3^ Owing to the extensive and varied benefits they offer, this practice is recognized and recommended globally in both industrialized and developing countries, thereby promoting improvements in the health of the mother-baby dyad.^2^ Breastfeeding should ideally be initiated within the first hour of life, ideally with skin-to-skin contact—the golden hour—and should be maintained exclusively for the first 6 months of the infant's life, making it the sole source of nutrition provided to the latent, with no other foods except vitamin drops or syrup supplements. The World Health Organization (WHO) also recommends that breastfeeding continue, alongside complementary foods, until the child is 2 years old or beyond, if the mother-baby dyad so desires.^1^^,^^4^ In Portugal, the General Directorate of Health maintains these recommendations. In 1994, the Baby-Friendly Hospital Initiative was implemented, aiming to promote and improve breastfeeding rates in Portugal.^5^ The biomechanics of suction during breastfeeding involve various orofacial structures. Several synchronized mandibular and lingual movements are performed, with the tongue being the fundamental organ for establishing an effective latch that allows the transfer of milk from the breast to the oral cavity. When the latch is maintained, there is no air inside the infant's oral cavity, and the tongue elevates the maternal nipple toward the hard palate, creating lateral channeling around the nipple (Figure 1). The soft palate remains close to the base of the tongue despite the anteroposterior movement of the tongue during suction.^6^ If the ventral surface of the tongue features a lingual frenulum with altered and restrictive anatomy, it may obstruct tongue movements and result in the congenital condition ankyloglossia (or tongue-tie). Ankyloglossia is a congenital alteration in tongue development, characterized by a short or thick lingual frenulum that limits its movements (partial ankyloglossia) or a tongue that appears to be fused to the floor of the mouth (total ankyloglossia).^7^

**Figure 1:**
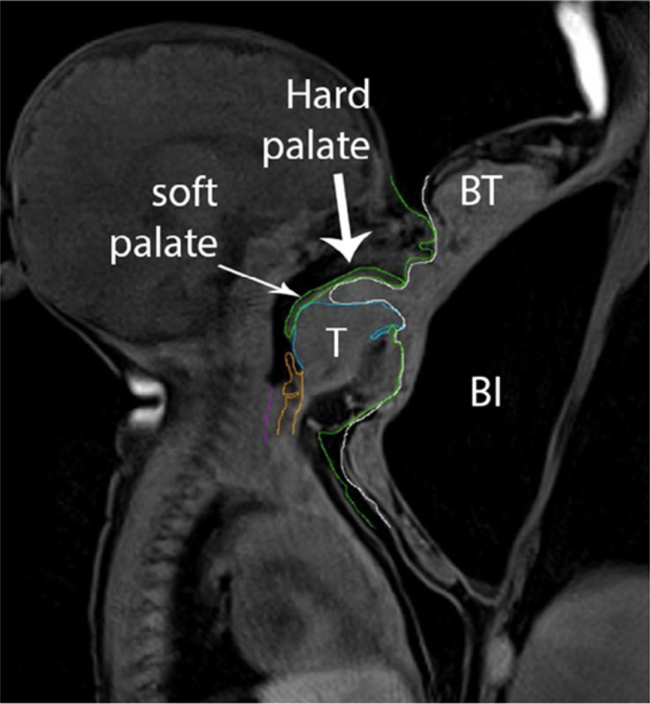
Suction biomecanics.^6^

The lingual frenulum is a flexible midline fold of fascia located beneath the oral mucosa in the anterior tongue. Its composition (layers) and points of attachment can vary significantly (Figure 2).^8^ Changes in the lingual frenulum can result in symptomatic ankyloglossia, characterized by the mother-infant dyad experiencing breastfeeding difficulties, or asymptomatic ankyloglossia, where no such issues are observed.^9^ Neonatal ankyloglossia in a breastfeeding infant can contribute to maternal nipple pain and trauma and hinder the infant's ability to effectively transfer breast milk, making it a potential risk factor for early discontinuation of breastfeeding.^10^^,^^11^ Cordray et al’s 2023 systematic review and meta-analysis concluded that pediatric ankyloglossia is commonly linked to challenges such as inadequate breastfeeding, infant gastroesophageal reflux, decreased maternal confidence in breastfeeding, and moderate nipple pain.^12^ Symptomatic ankyloglossia may discourage mothers from breastfeeding exclusively.^9^, ^10^, ^11^ Several functional and anatomical protocols are available to assist healthcare professionals in diagnosing neonatal ankyloglossia. However, there is a lack of standardization and consensus regarding these diagnostic criteria. Consequently, the prevalence of infant ankyloglossia remains uncertain and varies across countries, ranging from <1% to 20%.^13^ There are currently no scientific studies estimating the prevalence of ankyloglossia in Portugal and examining its correlation with functional impairment and breastfeeding-related pain. This study assessed the prevalence of neonatal ankyloglossia among Portuguese newborns and to evaluate whether this condition has influenced breastfeeding outcomes in these infants.

**Figure 2:**
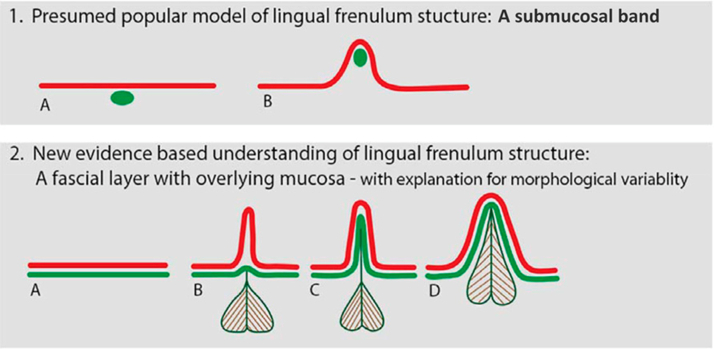
Anatomy of lingual frenulum.^8^

## Patients and Methods

A cross-sectional observational study was conducted involving neonates born at a central hospital in Oporto, Portugal, which reports approximately 2000 births annually. A sample comprising 25% of the annual newborn population was calculated, resulting in a total of 501 neonates. The sample size calculation considered estimations with 5% standard errors, a 2.0 design effect, 95% CIs, and a 10% potential nonresponse rate. A final sample of 501 infants was obtained. The prevalence of ankyloglossia in infants based on the combination of Assessment Tool for Lingual Frenulum Function (ATLFF) and Coryllos classification was set at 18%.^13^ The ATLFF was selected for diagnosing ankyloglossia, because it remains one of the most widely applied and structured instruments for newborn tongue-tie assessment, allowing evaluation of both the frenulum's anatomical appearance and tongue function during feeding. Data collection occurred between July 2023 and September 2024 and was conducted by a single investigator—a pediatric dentist and International Board-certified Lactation Consultant. This study was approved by the Health Ethics Committee of the São João University Hospital Center. The neonates were observed alongside their mothers during the first days of life, before discharge from the maternity ward. All participants received a participant information sheet detailing the study and provided written informed consent. There were no refusals or withdrawals during the investigation. Inclusion criteria for study participation required neonates to be healthy, born to mothers at a gestational age of ≥37 weeks, within the first 5 days of life, and with informed consent voluntarily provided and signed by their legal representative. The exclusion criteria included all individuals whose legal guardian was unable to respond to the presented questions; neonates diagnosed with (or under investigation for) any pathology, metabolic, genetic, or neurological disorders; clinical signs of illness; those who had undergone a lingual frenotomy; and any patient the investigator deemed ineligible for the study. All data were anonymized. After admitting study participants, general data regarding pregnancy and delivery were collected from clinical records, including the infant's gender, gestational age, type of delivery, any risk during pregnancy, birth weight, and Apgar score.

A questionnaire was administered to the mothers addressing several aspects of the postpartum period and breastfeeding, at the same time as the clinical assessment of the mother-infant dyad. Topics included skin-to-skin contact, the decision to breastfeed, breastfeeding within the first hour of a newborn's life, the current type of feeding (exclusive breastfeeding [EBF], mixed feeding, or exclusive formula feeding), the use of artificial nipples (nipple shields, bottles, and/or pacifiers), the presence of nipple trauma, and whether the mother experienced pain during breastfeeding. The breastfeeding session was observed by the examiner, and the mother's nipples were examined to assess the presence of cracks or nipple wounds. For mothers who did not report pain during breastfeeding, the questionnaire concluded at this point. For those who reported pain, additional questions were asked, including whether the pain was consistent throughout the breastfeeding session, whether they received support to correct the latch, and whether the pain was more pronounced in 1 breast. Subsequent questions addressed how the mother perceived the importance of the pain and its impact on her quality daily life.

The neonate's ankyloglossia was assessed using the Hazelbaker ATLFF^14^ in accordance with recommendations for the use of this protocol in newborns. When ankyloglossia was diagnosed, the lingual frenulum was classified according to 2015 Coryllos modified classification.^11^ The examiner underwent 2 sessions of training and calibration exercises for 4 hours following the ATLFF and Coryllos's criteria. Samples of 20 clinical case pictures were used to revise the neonate's frenulum. To obtain intraexaminer kappa values reliability, a 1-week interval between the 2 sessions was established. Intraexaminer kappa values were calculated considering each patient's final score for each criteria. The intraexaminer reliability values of Cohen's kappa agreement were 0.85 and 0.98, for ATLFF and Coryllos, respectively.

### Statistical Analyses

Statistical analysis was performed using STATA software, version 16.0 (Stata Corp). Initially, descriptive analyses of quantitative variables were conducted using measures of central tendency and dispersion (mean ± SD), and of categorical variables using absolute and relative frequencies. Subsequently, Poisson regression models with robust and adjusted variance estimates were applied to assess the association between EBF prevalence (EBFP) and related factors, including sociodemographic characteristics, pregnancy, delivery, postpartum data, breastfeeding practices, and the presence of ankyloglossia. Additionally, a Poisson regression analysis was performed to examine the association between the presence of pain during breastfeeding and the explanatory factors. Prevalence ratios (PRs) and their respective 95% CIs were used to estimate associations between the outcome variable and the independent variables. A *P* value of ≤20 in the unadjusted analysis was considered the criterion for including covariates in the adjusted regression model. In the adjusted models, covariate retention was based on a *P* value of ≤05.

## Results

This study included 501 newborns; 260 were male (52%) and 241 female (48%). The majority of the newborns (96%) were between 0 and 3 days of age at the time of study participation (n = 481), with only 20 (4%) being 4 or 5 days old. Regarding gestation, 395 postpartum women (78.9%) had a low-risk pregnancy corresponding with the newborn included in the study, whereas 21.1% (n = 106) experienced a high-risk pregnancy. Concerning the type of delivery, 50.5% of the newborns were delivered via spontaneous vaginal birth (n = 253), and 49.5% (n = 248) were delivered through noneutocic birth methods (cesarean section, forceps, vacuum extraction, or other). Birth weight was categorized into 3 groups based on the WHO classification: 30 newborns (6%) had low birth weight (<2500 g); the majority (n = 458 [91.4%]) had an appropriate weight for gestational age (between 2500 g and 4000 g); and 13 (5.6%) presented with high birth weight (≥4000 g). Within the first hour after birth, 421 newborns initiated breastfeeding (84%), and 408 experienced skin-to-skin contact with their mothers (81.5%). Most postpartum women (96%) expressed the intention to breastfeed their infants (n = 481). The prevalence of EBF during hospitalization was 71% (n = 355). The use of artificial nipples (silicone nipple shields, pacifiers, and/or bottles) was observed in 304 participants (60.7%). Specifically, pacifier use was reported in 41.7% of newborns (n = 209); 119 mothers (23.8%) used silicone nipple shields, and 30.3% of infants were bottle-fed (n = 152).

Regarding breastfeeding-related issues, 130 mothers (26%) reported experiencing some type of nipple trauma (such as wounds or fissures), and 28.4% (n = 142) reported nipple pain during breastfeeding. The duration of pain varied: 111 women (22.2%) indicated that the pain diminished over the course of the breastfeeding session, whereas 31 (6.2%) reported persistent pain throughout the session. Seventy-three mothers (14.7%) reported feeling more pain in 1 breast than the other, and 68 (13.7%) reported symmetrical pain in both breasts. When asked about their perception of the importance of pain during breastfeeding, 2 women (0.4%) stated that it was not important, 18 (3.5%) considered it of little importance, 19 (3.9%) said it was moderately important, and 38 (7.8%) thought it very important. Sixty-four women (12.8%) were unable to classify the importance of the pain. These results are summarized in Table I.

**Table I:** Characteristics of participants (n = 501)

Sociodemographic characteristics	No. (%)
Sex	
Male	260 (52)
Female	241 (48)
Infant age	
0-3 days old	481 (96)
4 or 5 days old	20 (4)
Pregnancy risk	
Low risk	395 (78.9)
High risk	106 (21.1)
Mode of delivery	
Eutocic	253 (50.5)
Noneutocic	248 (49.5)
Birth weight	
<2500 g	30 (6)
2500-4000 g	458 (91.4)
≥4000 g	13 (5.6)
Skin-to-skin contact immediately after birth	
No	93 (18.5)
Yes	408 (81.5)
Intention to breastfeed	
No	20 (4)
Yes	481 (96)
Initiation of breastfeeding within the first hour postpartum	
No	80 (16)
Yes	421 (84)
Exclusive maternal breastfeeding during the postpartum hospital stay	
No	146 (29)
Yes	355 (71)
Use of artificial nipples (silicone nipple shield, bottle, and/or pacifier)	
No	197 (39.3)
Yes	304 (60.7)
Use of pacifier	
No	292 (58.3)
Yes	209 (41.7)
Nipple trauma	
No	371 (74)
Yes	130 (26)
Breastfeeding pain	
No	359 (71.6)
Yes	142 (28.4)
Decreased pain during the feeding session	
No	31 (6.2)
Yes	111 (22.2)
Don't feel pain during breastfeeding	359 (71.6)
Importance of pain during breastfeeding	
Not important	2 (0.4)
Slightly important	18 (3.5)
Moderately important	19 (3.9)
Very important	38 (7.8)
Don't know	64 (12.8)
Don't feel pain during breastfeeding	359 (71.6)

The presence of neonatal ankyloglossia was identified in 13.8% of study participants (n = 69). Among the newborns diagnosed with ankyloglossia, the Coryllos classification of lingual frenulum was distributed as follows: 18.8% (n = 13) grade 1, 47.9% (n = 33) grade 2, 27.5% (n = 19) grade 3, 2.9% (n = 2) grade 4, and 2.9% (n = 2) grade 5. A greater prevalence of ankyloglossia was observed among male infants (58%) compared with females (42%), but with no statistical significance. The results related to ankyloglossia diagnosis are summarized in Table II.

**Table II:** Clinical assessment of the lingual frenulum of newborns (n = 501)

Clinical assessment of the lingual frenulum	No. (%)
Presence of ankyloglossia (according to the ATLFF) (n = 501)	
No	432 (86.2)
Yes	69 (13.8)
Modified Coryllos classification in presence of ankyloglossia (n = 69)	
Grade 1	13 (18.8)
Grade 2	33 (47.9)
Grade 3	19 (27.5)
Grade 4	2 (2.9)
Grade 5	2 (2.9)
Gender with ankyloglossia (n = 69)	
Male	40 (58)
Female	29 (42)

Table III presents the association between the EBFP during hospitalization and associated factors. The adjusted Poisson regression model showed a lower likelihood of EBFP among dyads with high-risk pregnancies (PR, 0.82; 95% CI, 0.71-0.94; *P* = .006). Women who expressed a desire to breastfeed had a greater likelihood of achieving EBF during the postpartum hospital stay (PR, 1.68; 95% CI, 1.00-2.82; *P* = .049), as did those who initiated breastfeeding within the first hour of life (PR, 1.51; 95% CI, 1.15-1.98; *P* = .003). The presence of artificial nipple use was associated with a lower likelihood of EBF (PR, 0.58; 95% CI, 0.52-0.64; *P* < .001). Maternal nipple trauma was initially associated with EBF in the unadjusted robust model; however, this association was not maintained after adjustment. Infant age, type of delivery, skin-to-skin contact, breastfeeding-related pain, and the presence of ankyloglossia were not associated with EBFP.

**Table III:** Assessment of factors associated with EBFP before hospital discharge

Associated factors	Unadjusted analysis	Adjusted analysis
	PR (95% CI)	PR (95% CI)
Infant age		
0 Eutocic 3 days old	1	
4 or 5 days old	0.99 (0.74-1.32)	
Pregnancy risk		
Low risk	1	1
High risk	0.78 (0.65-0.92)	0.82 (0.71-0.94)
Mode of delivery		
Eutocic	1	
Noneutocic	0.89 (0.80-1.00)	
Skin-to-skin contact immediately after birth		
No	1	
Yes	1.09 (0.94-1.29)	
Intention to breastfeed		
No	1	1
Yes	2.42 (1.24-4.74)	1.68 (1.00-2.82)
Initiation of breastfeeding within the first hour postpartum		
No	1	1
Yes	1.98 (1.50-2.63)	1.51 (1.15-1.98)
Use of artificial nipples (silicone nipple shield, bottle, and/or pacifier)		
No	1	1
Yes	0.54 (0.48-0.59)	0.58 (0.52-0.64)
Nipple trauma		
No	1	
Yes	0.89 (0.77-1.02)	
Breastfeeding pain		
No	1	
Yes	0.94 (0.82-1.07)	
Presence of ankyloglossia (according to the ATLFF)		
No	1	
Yes	1.03 (0.88-1.20)	

*PR*, prevalence ratio.

Table IV presents the results for the presence of pain during breastfeeding and associated factors. The adjusted Poisson regression model indicates a lower likelihood of maternal pain during breastfeeding among newborns between 4 and 5 days old compared with the youngest ones (PR, 0.31; 95% CI, 0.09-0.97; *P* = .045). Mothers of newborns with ankyloglossia had a significantly higher probability of experiencing breastfeeding-related pain (PR, 1.51; 95% CI, 1.11-2.06; *P* = .009), as did those presenting with nipple trauma (PR, 3.24; 95% CI, 2.47-4.25; *P* < .001). In the initial unadjusted analysis, the use of artificial nipples was associated with the presence of breastfeeding-related pain (PR, 1.49; 95% CI, 1.09-2.03; *P* = .011); however, this association was not observed in the adjusted model (PR, 0.31; 95% CI, 0.09-1.74; *P* = .071). A similar pattern was observed for the type of delivery. An initial association between noneutocic birth and breastfeeding pain was not maintained in the adjusted model. Desire to breastfeed, initiation of breastfeeding within the first hour after birth, and EBF during hospitalization were not associated with the presence of maternal nipple pain during breastfeeding.

**Table IV:** Assessment of factors associated with Maternal Breastfeeding Pain

Associated factors	Unadjusted analysis	Adjusted analysis
	PR (95% CI)	PR (95% CI)
Infant age		
0-3 days old	1	1
4 or 5 days old	0.34 (0.09-1.29)	0.31 (0.09-0.97)
Mode of delivery		
Eutocic	1	
Noneutocic	0.75 (0.56-0.99)	
Intention to breastfeed		
No	1	
Yes	1.44 (0.59-4.49)	
Initiation of breastfeeding within the first hour postpartum		
No	1	
Yes	1.31 (0.85-2.02)	
Exclusive maternal breastfeeding during the postpartum hospital stay		
No	1	
Yes	0.86 (0.64-1.15)	
Use of artificial nipples (silicone nipple shield, bottle, and/or pacifier)		
No	1	1
Yes	1.49 (1.09-2.03)	1.30 (0.97-1.74)
Nipple trauma		
No	1	1
Yes	3.48 (2.67-4.53)	3.24 (2.47-4.25)
Presence of ankyloglossia (according to the ATLFF)		
No	1	1
Yes	1.82 (1.35-2.46)	1.51 (1.11-2.06)

*PR*, prevalence ratio.

## Discussion

The Global Breastfeeding Initiative in Hospitals (GBIH), launched in 1991 following the Innocenti Declaration, aims to implement evidence-based practices that protect, promote, and support breastfeeding worldwide, recognizing its crucial role in improving maternal and infant health outcomes.^15^ Introduced in Portugal in 1994, the initiative coincided with a rise in EBFP at 6 months from 20.6% in 1995 and 1996 to 30.3% in 2014.^5^ Despite this progress, several Portuguese studies have reported lower-than-targeted EBF rates: a 2019 study in Lisbon found an EBFP of 74.2% at discharge, dropping to 25.6% at 6 months^16^; another study in Porto observed rates of 80.5% at discharge and 59.7% at 2 months^17^; and a 2022 national study reported 21.8% EBF at 6 months,^18^ still below the World Health Assembly goal of 50% by 2025.^19^ More recently, an online cross-sectional study conducted in Portugal between 2020 and 2022 reported an EBFP of 64.5% and a pediatric ankyloglossia prevalence of 16%, with no observed association between ankyloglossia and EBF rates.^20^ The breastfeeding experience can be significantly impacted for mothers of infants with ankyloglossia. This condition, characterized by restricted tongue mobility, is often associated with challenges such as difficulty achieving a proper latch, ineffective sucking, reduced milk transfer, disruptions in the milk ejection reflex, and insufficient infant weight gain.^21^^,^^22^ In a recent cross-sectional study, Campanha et al demonstrated that infants with ankyloglossia are 36.07 times more likely to encounter breastfeeding challenges than those without, with a particular emphasis on difficulties related to effective sucking.^23^ Riskin et al highlighted that infants with tongue-tie, whether presenting as anterior or posterior ankyloglossia, are more likely to face significant breastfeeding difficulties during the first 30 days of life.^24^ A recent multicenter cohort study found no significant differences in exclusive or total breastfeeding rates between infants with and without ankyloglossia at 1, 4, and 6 months. The authors noted that the low prevalence of ankyloglossia and high loss to follow-up limited the strength of their conclusions. They also cautioned against overinterpreting the results and acknowledged that a potential impact of ankyloglossia on breastfeeding difficulties cannot be ruled out.^25^

The variations in the reported prevalence of ankyloglossia in infants in recent years may be linked to the lack of consensus about diagnostic criteria and protocols. Compared with 10 or 20 years ago, the diagnosis of ankyloglossia has increased significantly, likely owing to growing awareness of its potential impact on breastfeeding. Today, ankyloglossia is the second most frequently discussed topic in online groups and social media platforms focused on breastfeeding.^26^ The systematic review and meta-analysis by Cruz et al (2023) on the prevalence of ankyloglossia includes various diagnostic methods and classification systems, such as the Coryllos protocol and the ATLFF.^13^ Owing to methodological differences, prevalence rates vary widely, and there is no consensus on the most appropriate diagnostic approach. The meta-analysis found an overall ankyloglossia prevalence of 5% across infants, children, and adolescents. Among infants specifically, the prevalence was 7%. Higher rates were observed when using the Coryllos protocol alone (20%), with slightly lower rates when combining the Coryllos and ATLFF classifications (18%). The authors also noted a publication year effect, with 66.2% of included studies published from 2011 onward, suggesting increased recognition of ankyloglossia in recent years.^13^

The present study found a neonatal ankyloglossia prevalence of 13.8%, slightly higher than most figures reported in the literature. Our sample showed a higher distribution of ankyloglossia among type 2 lingual frenulum, according to the Coryllos classification, whereas in Maya-Enero et al (2021), most cases were classified as type 3.^27^ One likely explanation for the lower prevalence rates reported in the literature is that Coryllos type 1 and type 2 frenulum are more easily identified than the more posterior or submucosal types (types 3, 4, and 5 in the Coryllos classification). This diagnostic difficulty may contribute to the underdiagnosis of ankyloglossia in study populations. However, Ghaheri et al reported a greater prevalence of posterior lingual frenulum compared with anterior ones (78% vs 21%), a finding consistent with the results reported in Maya-Enero et al.^27^^,^^28^ A regression analysis of our sample showed no statistically significant association between the diagnosis of ankyloglossia and the prevalence of EBF. However, a positive association was found between ankyloglossia and maternal nipple pain during breastfeeding, suggesting that the presence of ankyloglossia in infants may increase maternal discomfort during breastfeeding. In its 2021 position statement on ankyloglossia in breastfeeding dyads, the Academy of Breastfeeding Medicine highlights neonatal ankyloglossia as a potential contributing factor to early weaning, owing to associated nipple pain, trauma, and ineffective milk transfer by the infant.^10^ Our results align with previous studies suggesting that restricted tongue mobility may interfere with effective latching and milk transfer, contributing to nipple trauma and maternal discomfort. Additionally, maternal pain was more likely when newborns were <4 days old, which may reflect the initial challenges of breastfeeding adaptation in the early postpartum period. This critical window may increase sensitivity to breastfeeding, because both the infant and mother are establishing feeding techniques. The strong association between nipple trauma and pain further underscores the need for early assessment and intervention to prevent complications that may compromise breastfeeding.

Regarding EBF, our investigation revealed dyads from low-risk pregnancies were significantly more likely to achieve EBFP, suggesting that fewer maternal or neonatal complications may facilitate early breastfeeding success. This finding is consistent with previous literature, emphasizing the positive impact of uncomplicated pregnancies on breastfeeding outcomes. Maternal intention to breastfeed emerged as a strong predictor of EBFP, and women who expressed a desire to breastfeed were significantly more likely to do so exclusively during hospitalization. This finding underscores the importance of prenatal counseling and support, because maternal motivation plays a crucial role in the initiation and continuation of breastfeeding. Early initiation of breastfeeding (within the first hour of life) was also positively associated with EBFP, reinforcing WHO recommendations. Furthermore, the absence of artificial nipples was strongly associated with higher rates of EBF. These findings suggest that behavioral and attitudinal factors, such as maternal intention and feeding practices, may have a greater influence on early EBF.

A limitation of this study is that all assessments were performed by a single examiner. This approach was necessary, because no other professionals in Oporto (Portugal) possessed the combined expertise required to conduct standardized tongue-tie evaluations using the selected protocol and to address breastfeeding issues. Although this factor ensured methodological consistency, it may limit the generalizability of the findings to other settings or institutions. Future multicenter studies involving multiple trained examiners across different hospitals are warranted to validate and extend these results.

## Conclusions

This study contributes to a deeper understanding of the factors influencing EBF and maternal breastfeeding-related pain in the early postpartum period. Although no significant association was found between ankyloglossia and EBF, its presence was positively associated with maternal nipple pain, highlighting the potential impact of tongue-tie on breastfeeding comfort rather than feeding outcomes. These findings underscore the importance of promoting early breastfeeding practices and addressing anatomical and behavioral challenges to support successful and comfortable breastfeeding experiences, as well as the implementation of routine lingual frenulum evaluation before hospital discharge.

## Declaration of Generative AI and AI-assisted Technologies in the Writing Process

During the preparation of this work, the authors used ChatGPT 4 to improve readability and language. After using this tool, the authors reviewed and edited the content as needed and take full responsibility for the content of publication.

## CRediT authorship contribution statement

**J. Morgado Dias:** Writing – review & editing, Writing – original draft, Visualization, Validation, Supervision, Software, Resources, Project administration, Methodology, Investigation, Formal analysis, Data curation, Conceptualization. **J. Abanto:** Writing – review & editing, Writing – original draft, Visualization, Validation, Supervision, Software, Resources, Project administration, Methodology, Investigation, Formal analysis, Data curation, Conceptualization. **J. Correia Pinto:** Writing – review & editing, Supervision, Methodology, Formal analysis, Data curation, Conceptualization. **C. Areias:** Writing – review & editing, Supervision, Methodology, Data curation. **H. Soares:** Writing – review & editing, Writing – original draft, Visualization, Validation, Resources, Project administration, Methodology, Investigation, Conceptualization.

## Declaration of Competing Interest

The authors declare no conflicts of interest.
